# Dedifferentiation and Transfer in Executive Function and Math Ability Following a Five‐Year Abacus Training in Schoolchildren

**DOI:** 10.1002/advs.202504518

**Published:** 2025-07-11

**Authors:** Tianyong Xu, Xinyang Liu, Hongjian He, Changsong Zhou, Andrea Hildebrandt, Feiyan Chen

**Affiliations:** ^1^ School of Physics Zhejiang University Hangzhou 310058 China; ^2^ Department of Radiology and Biomedical Imaging University of California San Francisco San Francisco California 94158 USA; ^3^ Key Laboratory of Brain Functional Genomics (MOE & STCSM) Affiliated Mental Health Center (ECNU) Institute of Brain and Education Innovation School of Psychology and Cognitive Science East China Normal University Shanghai 200062 China; ^4^ Department of Physics Centre for Nonlinear Studies Institute of Computational and Theoretical Studies Hong Kong Baptist University Hong Kong China; ^5^ Life Science Imaging Centre Hong Kong Baptist University Hong Kong China; ^6^ Department of Psychology School of Medicine and Health Sciences Carl von Ossietzky Universität Oldenburg 26129 Oldenburg Germany; ^7^ Research Center Neurosensory Science Carl von Ossietzky Universität Oldenburg 26129 Oldenburg Germany

**Keywords:** cognitive training, dedifferentiation, executive function, functional connectivity, individual difference, mathematical ability

## Abstract

The cognitive differentiation of executive function (EF) and mathematical ability during child development, characterized by their decreasing correlation, is well established. However, the impact of long‐term cognitive training on this developmental effect remains largely unexplored. The present study investigated this by analyzing behavioral and neuroimaging data from schoolchildren who participated in five years of abacus training. The findings indicate that, compared to the control group, the training group exhibits cognitive dedifferentiation, characterized by stronger correlations between EF and mathematical abilities, accompanied by lower inter‐individual variability. These observations are consistent with the discovery of greater overlap in behavior‐associated brain connectivity patterns and more uniform connectivity profiles across individuals. Furthermore, the individual‐to‐group similarity in connectivity pattern is significantly associated with EF and mathematical performance, suggesting a shared cognitive strategy shaped by prolonged training. The findings provide empirical evidence in support of neurocognitive plasticity, highlighting the capacity of targeted cognitive training to functionally reshape brain networks and modulate the developmental trajectory of cognitive traits.

## Graphical abstract

While cognitive development typically exhibits differentiation, marked by decreasing inter‐domain correlations, this study demonstrates that five‐year abacus training is associated with de‐differentiation of executive function and mathematical ability in school‐aged children. This is evidenced by stronger inter‐domain correlations and greater integration of behavior‐associated networks, revealing neurocognitive plasticity through target training.

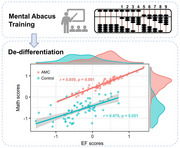

## 1 Introduction

Human cognitive abilities form a dynamic, interconnected system that continuously adapts across the lifespan. Cognitive developmental theory examines how the structure of cognition evolves through age‐related and experience‐dependent learning.^[^ ^1^, ^2^, ^3^, ^4^ ^]^ This often manifests as either differentiation, which is characterized by an increase in specialization and decreased inter‐correlations among cognitive domains, or dedifferentiation, which is characterized by greater overlap and increased inter‐correlations. Among these domains, executive function (EF) and mathematical ability play pivotal roles in shaping children's academic achievement and long‐term developmental outcomes.^[^ ^5^, ^6^ ^]^ However, the impact of targeted cognitive training on the development and interaction of these abilities in school‐aged children, along with their neural underpinnings, remains complex and has not yet been sufficiently explored.

During development, children typically exhibit increasing cognitive specialization, with declining correlations between distinct abilities as they mature.^[^ ^1^, ^2^ ^]^ This process, known as differentiation, aligns with Spearman's law of diminishing returns, suggesting that younger and less‐skilled children rely more on EF for mathematical tasks, whereas older or more proficient individuals increasingly employ procedural skills and retrieval strategies.^[^ ^5^, ^7^, ^8^, ^9^, ^10^, ^11^, ^12^, ^13^ ^]^ Consistent with these findings, neurobiological evidence demonstrates that cognitive differentiation is associated with increased modularity in brain networks, as seen in typical developmental progressions. In contrast, cognitive dedifferentiation tends to correspond with reduced modularity, a pattern frequently observed during aging.^[^ ^1^, ^14^, ^15^, ^16^, ^17^, ^18^ ^]^ Training interventions during development can modulate this dynamic process in complex ways. In this regard, a training paradigm targeting multiple distinct abilities may enhance their correlation through transfer effects; while individuals with initially weaker abilities may show greater improvements, potentially reducing these correlations between the trained ability and other cognitive abilities through a compensatory effect. This raises the question of whether specific training promotes the differentiation or dedifferentiation of these cognitive abilities, along with the nature of the brain reorganization that occurs.

Cognitive training has been demonstrated to influence brain function and cognitive abilities^[^ ^19^, ^20^ ^]^ while this field of research continues to be marked by ongoing debates. A contentious issue is the concept of “far transfer,” which posits that improvements in performance on trained tasks can generalize to untrained tasks.^[^ ^21^, ^22^, ^23^, ^24^ ^]^ In addition, recent empirical research reported in the *Journal of Nature Neuroscience* suggests that eight weeks of cognitive control training focused on response inhibition had no effect on neural or behavioral outcomes in children.^[^ ^25^ ^]^ The efficacy and multifaceted impacts of cognitive training are contingent upon a multitude of factors, including the specific strategies employed, the skills targeted, the duration of the training, the pedagogical methods utilized, and the socioeconomic status and age of participants.^[^ ^20^ ^]^

It is imperative that well‐designed training is provided in order to further understand the aforementioned issue. An abacus mental calculation (AMC) program provides an extensive training regimen over the long term for schoolchildren.^[^ ^26^, ^27^ ^]^ First, while short‐term training typically lasts weeks or months, this multi‐year regimen may induce lasting neurocognitive changes by leveraging brain plasticity to preserve training benefits. Moreover, this program targets multiple cognitive domains, focusing on enhancing mathematical ability and EF through a structured visuospatial approach to mental arithmetic.^[^ ^28^, ^29^, ^30^ ^]^ As demonstrated in previous AMC studies, training‐induced changes in brain function have been observed in fronto‐parietal regions, which are closely linked to EF and mathematical ability.^[^ ^31^, ^32^, ^33^, ^34^, ^35^ ^]^ Thus, long‐term AMC training provides a unique opportunity to study how cognitive training shapes both cognitive structures and their neural underpinnings beyond typical developmental trajectories.

The developmental differentiation of cognitive abilities has been intrinsically associated with individual variations in cognitive and neurobiological maturation.^[^ ^36^, ^37^ ^]^ Notably, the extent of this differentiation varies according to individual proficiency levels across different cognitive domains, as characterized by distinct neural markers.^[^ ^38^, ^39^ ^]^ Training has the potential to shape individual differences in brain function and cognitive behavior, particularly through sustained, deliberate practice that fosters expert performance.^[^ ^40^, ^41^, ^42^ ^]^ Empirical studies indicate that intensive cognitive training can lead to reorganizations of functional brain networks.^[^ ^43^, ^44^, ^45^ ^]^ Furthermore, consistent cognitive strategies applied when performing tasks belonging to different cognitive domains may further reduce the variability in cognitive traits and brain functions between individuals.^[^ ^41^, ^46^ ^]^ Additionally, inter‐individual similarities in cognitive traits correlate with aligned functional patterns in relevant neural systems.^[^ ^47^, ^48^, ^49^ ^]^ This evidence suggests that shared characteristics of cognitive abilities may arise from analogous learning experiences, as reflected in corresponding neural representations.^[^ ^50^, ^51^ ^]^

Given the unique visuospatial strategy applied in AMC training and its close association with EF and mathematical ability, we expected that prolonged AMC training might yield unique cognitive and neural outcomes, potentially modifying the typical developmental differentiation between EF and mathematical ability observed in childhood. To test this hypothesis, we analyzed behavioral and neuroimaging data from school‐aged children after five years of AMC training (see Experimental Section). First, latent variable analysis revealed stronger, more consistent ability associations in AMC‐trained children in comparison to the control group. Second, functional connectivity (FC) analyses within fronto‐parietal regions demonstrated higher network pattern overlap between EF and math‐related networks, along with greater inter‐individual similarity of connectivity profile in post‐training. Finally, within the trained group, a significant correlation was identified between connectivity architecture similarity and cognitive performance scores. Taken together, the findings reveal a significant pattern of training‐induced cognitive dedifferentiation effect and its neural correlates. This is characterized by stronger inter‐correlations between cognitive abilities, higher overlap with shared neural systems, and greater inter‐individual similarity of connectivity profile associated with individual cognitive performance. These findings provide crucial insights into how cognitive training may reshape the underlying cognitive structure and neural organization, indicating a more comprehensive and interconnected impact on cognitive functioning than previously recognized.

## 2 Results

We first analyzed the behavioral data pertaining to mathematical and EF abilities, which were collected from school‐aged children in the training group after five years of training, as well as from their control peers. Our findings demonstrated that the AMC training group outperformed the control group significantly in both EF and mathematical abilities (see Figure S1, Supporting Information). By focusing on these fundamental aspects of EF and mathematical ability, we aimed to identify the training‐induced disparities in both the inter‐domain relationship in cognition and the inter‐individual variability of these abilities between training and control groups.

### 2.1 Inter‐Domain Relationship and Inter‐Individual Variability of EF and Mathematical Ability

**Figure 1:**
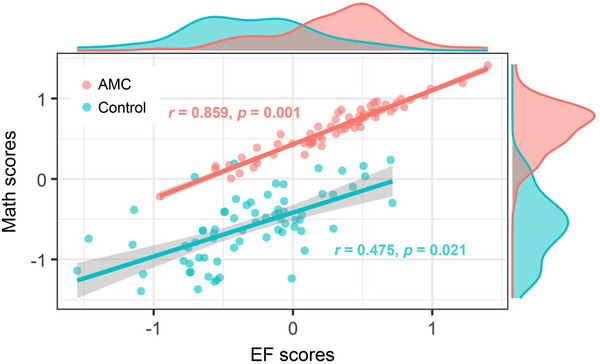
Dedifferentiation of the inter‐domain relationship in cognition and differences in the inter‐individual variability of EF and mathematical ability in the AMC group compared to the control group (*n* = 141, covariance analysis in SEM, all *p* < 0.05). To illustrate these findings, the latent variable derived scores for EF and mathematical ability, as estimated in Model 2, are displayed in a scatter plot.

We performed a stepwise multi‐group structural equation modelling (SEM) analysis (see Experimental Section). Utilizing cognitive indicators from both groups, a basic model was first constructed to assess the factorial structure of the EF and mathematical ability assessments included in this study. Subsequent to this, model comparison tests were performed to assess measurement invariance across the groups through the implementation of a stepwise procedure of model constraints. The results of model comparison are shown in **Table** **1**, involving three specific tests: Model 1 versus Model 2, Model 2 versus Model 3, and Model 3 versus Model 4. The unconstrained Model 1–which allowed all model parameters to be group specific–had an acceptable fit: *χ* ^2^(24) = 54.242, *p* = 0.000, CFI = 0.938, RMSEA = 0.134, SRMR = 0.082. Model 2, which constrained factor loadings to be equal across groups, did not differ significantly from the unconstrained Model 1 in terms of fit (Δ*χ* ^2^ = 4.545, *p* = 0.474), indicating an invariant metric of measurement of the latent abilities across groups. Descriptively, the model results revealed a stronger correlation between EF and mathematical ability (training group: *r* = 0.859, *p* = 0.001; control group: *r* = 0.475, *p* = 0.021), as well as lower individual variance in these abilities within the AMC group compared to the control group (**Figure** **1**).

**Table 1:** Tests of model comparison in multiple group SEM analysis.

Model	df	AIC	BIC	χ^2^	Δχ^2^	RMSEA	Δdf	*p*(Δχ^2^)
Model 1: unconstrained model	24	2100.1	2235.8	54.242				
Model 2: FL	29	2094.7	2215.6	58.787	4.545	0.000	5	0.474
Model 3: FL+lv.VAR	31	2097.6	2212.6	65.693	6.906	0.187	2	0.032*
Model 4: FL+lv.VAR+lv.COVAR	32	2102.9	2215.0	73.033	7.340	0.300	1	0.007**

Model description: Model 1 is unconstrained. All model parameters are group‐specific. In Model 2, factor loadings are constrained to be equal across groups. In Model 3, both factor loadings and the latent variable variances are constrained to be equal across groups. In Model 4, factor loadings, latent variable variances, and covariances are constrained to be equal across groups. *Abbreviations*: FL, factor loadings; lv.VAR, variance of latent variables; lv.COVAR, covariance between the two latent variables. Note: ^***^ *p* < 0.001; ^**^ *p* < 0.01; ^*^ *p* < 0.05.

Factor structure invariance was further tested through a step‐wise model comparison, specifically between Model 2 and Model 3, as well as between Model 3 and Model 4. In Model 3, both factor loadings and latent variable variances were constrained to be equal across groups. The comparison between Model 3 and Model 2 revealed a significant difference (Δ*χ* ^2^ = 6.906, *p* = 0.032), indicating that the assumption of equal variances across groups did not hold. This finding suggests significantly lower individual variability in latent EF and mathematical abilities within the AMC group (Var _EF_ = 0.236, Var _Math_ = 0.092), compared to the control group (Var _EF_ = 0.376, Var _Math_ = 0.176), supporting the hypothesis that the training intervention led to more consistent performance in these cognitive domains among AMC trained individuals. Model 4, which further constrained the latent variable covariances to be equal across groups, showed a significant difference as compared with Model 3 (Δ*χ* ^2^ = 7.340, *p* = 0.007). This result indicates that the relationship between EF and mathematical ability differs significantly between the AMC group and the control group. Specifically, this suggests that the training group exhibited a stronger association between EF and mathematical ability, highlighting a reshaped organization of these cognitive domains as a result of the intervention.

In summary, the results of the model comparisons revealed two key findings: 1) the training group exhibited lower individual variability in EF and mathematical ability, as evidenced by the comparison between Model 2 and Model 3, and 2 the training group demonstrated a stronger relationship between EF and mathematical ability, as shown by the significant difference between Model 3 and Model 4. For additional illustration, the observed pairwise correlations between EF and mathematical ability indicators for the AMC group, compared to the control group, is provided in the Figure S2, Supporting Information.

### 2.2 Group Differences in FC Value

**Figure 2:**
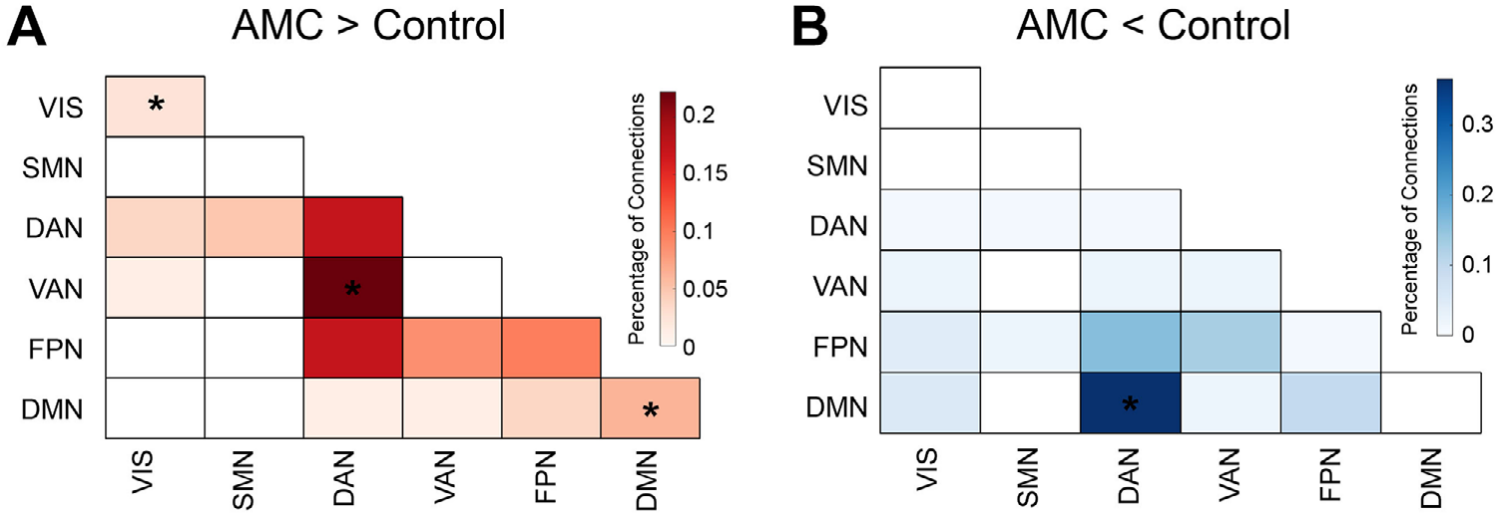
FCs with group differences. Network distribution of FCs with (A) stronger value or (B) weaker value in the AMC group compared to the control group (*n* = 59, permutation test, corrected *p* < 0.05). Note: the asterisk (*) denotes the cells (within/between networks) with considerable FCs with significant group difference after correction (permutation test, corrected *p* < 0.05). *Abbreviations*: VIS, visual network; SMN, sensory/motor network; DAN, dorsal attention network; VAN, ventral attention network; FPN, frontoparietal network; DMN, default mode network.

Given the observed group differences in cognitive performance and their established association with fronto‐parietal regions, we examined between‐group differences in FC within these regions using a permutation test (see Experimental Section). The analysis revealed that, compared to controls, the AMC group exhibited significantly stronger FC in several networks (**Figure** **2**): most prominently between the dorsal attention network (DAN) and ventral attention network (VAN), as well as within the default mode network (DMN) and visual network (VIS) (permutation test, corrected *p* < 0.05). A trend‐level of stronger connectivity was also observed within DAN, as well as between DAN and the fronto‐parietal network (FPN). Conversely, the AMC group showed significantly weaker FC between DMN and DAN (corrected *p* < 0.05). Spatially, these FCs were mainly distributed between the middle/inferior frontal gyrus and the posterior lateral parietal cortex (Figure S3, Supporting Information).

### 2.3 Connectivity Pattern Similarity Across Cognitive Domains

**Figure 3:**
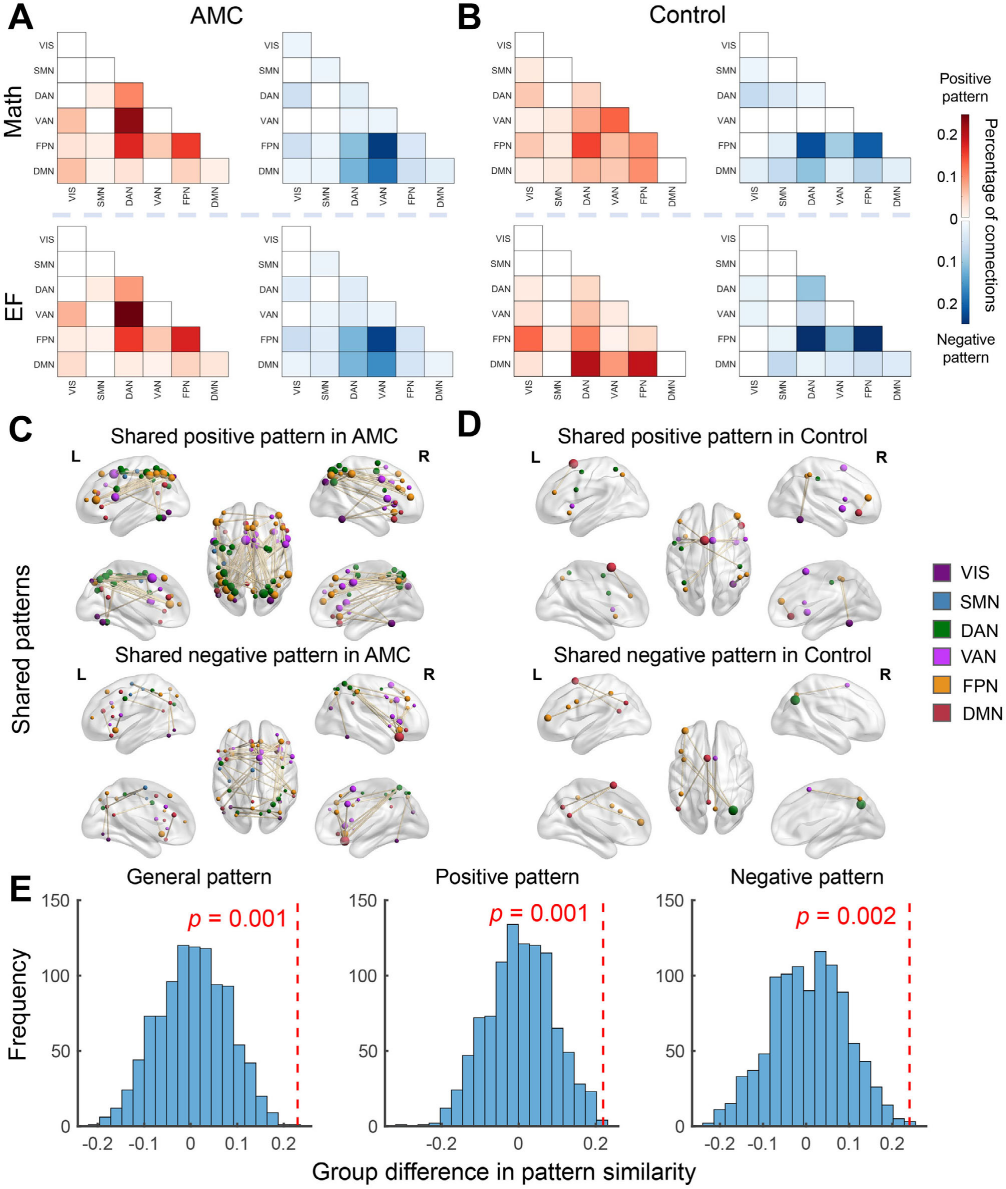
Behavior‐related connectivity patterns in the domain of EF and mathematical ability (abbreviated Math in the figure) and their similarity within groups. (A,B) EF‐ and mathematical ability‐related connectivity patterns in the AMC and control groups are visualized, with color shading indicating the proportion of behavior‐related FCs within or between networks. Warm colors represent positive correlations, and cold colors represent negative correlations. (C,D) Visualization of overlapping FC patterns between EF and mathematical processing in training and control cohorts. Node dimensions reflect degree centrality, quantified as the total number of functional connections per node. (E) The training group showed significantly higher behavior‐related pattern similarity between EF and mathematical ability than the control group (*n* = 59, permutation test, all *p* < 0.005; red dashed line = observed data). General similarity reflects the average similarity of positive and negative functional connections, resulting from separate analyses for each.

To quantify the shared connectivity architecture between EF and mathematical ability, we first identified relevant connections for each ability domain (see Experimental Section). This yielded both positive and negative FC patterns for each ability in each group (**Figure** **3**A,B). In the AMC group, behavior‐related FCs of the positive patterns for EF and mathematical ability were primarily distributed in the DAN, FPN, and VAN, while negative patterns were observed in the VAN, DMN, and FPN (Figure 3A). In the control group, positive patterns were mainly distributed across the DAN, DMN, and FPN, while negative patterns were found between FPN and DAN, as well as within FPN (Figure 3B).

Subsequent analyses examining behavior‐shared connectivity patterns within each group revealed significantly greater pattern similarity in the training group compared to the control group, with quantitative assessment of behavior‐related connectivity patterns demonstrating distinct organizational patterns. As depicted in Figure 3C,D, the training group exhibited greater shared functional connections, particularly manifesting in dense fronto‐parietal region interactions, while the control group displayed markedly sparse shared functional connections across these regions. We employed a normalized angle metric to quantitatively assess the similarity between behavior‐associated connectivity patterns related to EF and mathematical ability within each group. Specifically, the training group demonstrated significantly higher pattern similarity indices, with positive pattern similarity between EF‐ and mathematics‐related connectivity patterns reaching 0.793, and negative pattern similarity achieving 0.751. These indices were notably higher than those observed in the control group, which exhibited significantly lower indices (positive pattern similarity: 0.573; negative pattern similarity: 0.511).

Furthermore, this group difference in pattern similarity was statistically analyzed by a permutation test (Figure 3E; see Experimental Section). The null hypothesis, which posited no difference in similarity for positive and/or negative FC patterns between groups, was tested using a permutation test with 1000 random shuffles of group labels. The AMC group exhibited a significantly higher degree of similarity between EF and mathematical ability patterns, as reflected in the general pattern (*p* = 0.001), which included both positive and negative FC patterns, as well as in the separate positive pattern (*p* = 0.001) and separate negative pattern (*p* = 0.002). The findings indicate a greater degree of overlap in connectivity patterns between EF and mathematical ability, relevant connections in the AMC group, which lends support to the stronger link between these abilities following long‐term AMC training.

In addition, it is imperative to underscore the observed network‐level consistency between the distribution patterns of FC associated with group differences and the behavior‐related connectivity patterns. Specifically, the distribution of higher FC values within the training group (Figure 2A) closely parallels the positively correlated behavioral connectivity patterns identified in the same group (Mathematical ability: *r* = 0.873, *p* < 0.001; EF: *r* = 0.866, *p* < 0.001; Figure 3A). And the distribution of higher FC values within the control group (Figure 2B) exhibits a markedly similar pattern to the EF‐related connectivity patterns observed in that group (Mathematical ability: *r* = 0.399, *p* = 0.073; EF: *r* = 0.731, *p* < 0.001; Figure 3B). This finding indicates that the training‐induced changes in FC may correspond with cognition‐specific connectivity patterns, highlighting the distinct neural reorganization processes that emerge as a result of training. The training group appears to show a shift toward connectivity patterns that support cognition‐specific improvements, while the control group maintains a stronger emphasis on EF‐related connectivity. This dynamic pattern highlights the potential for targeted training to selectively modulate FC in ways that are aligned with specific cognitive outcomes.

### 2.4 Inter‐Individual Similarity of Connectivity Architecture

**Figure 4:**
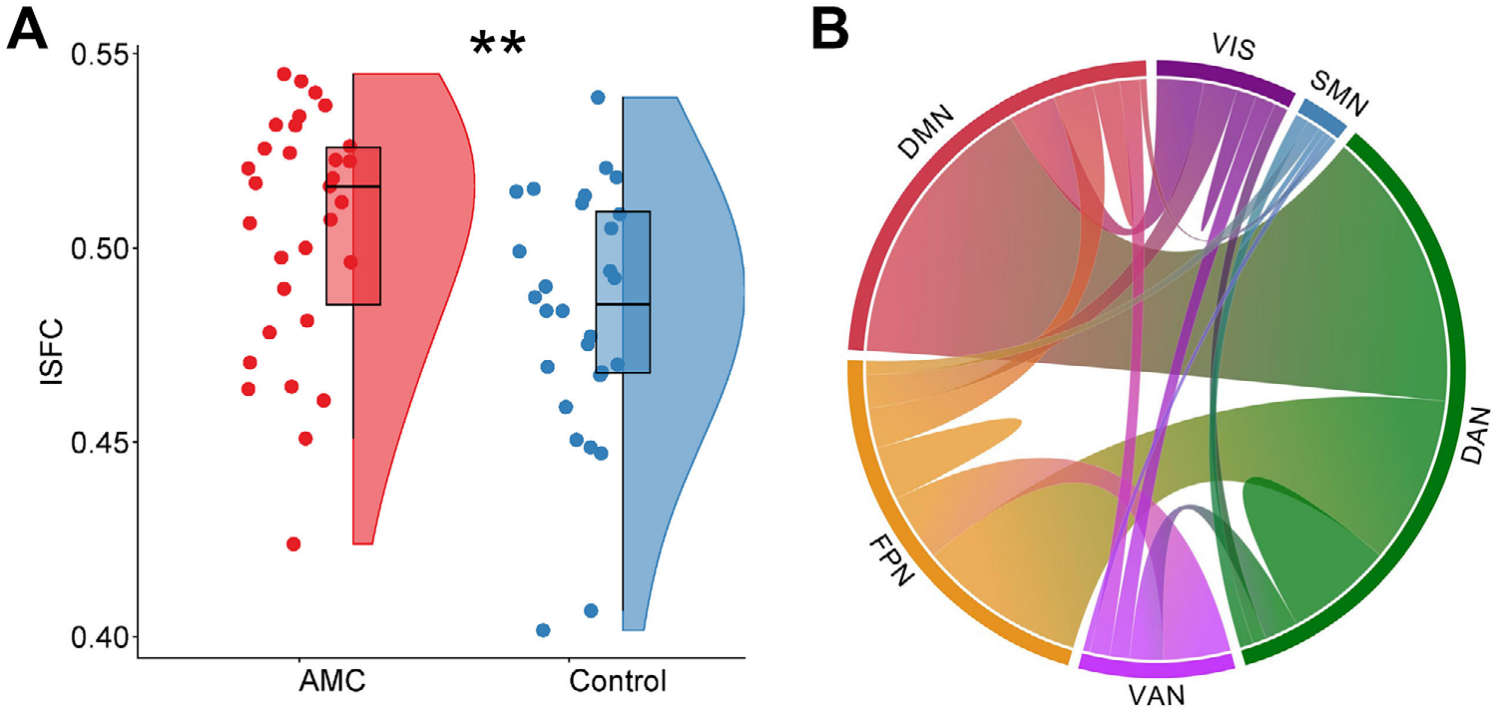
Individual ISFCs and critical FCs. (A) The AMC group exhibited higher ISFC compared to the control group (*n* = 59, independent sample *t*‐test, ^**^ *p* < 0.01). B) Distribution of critical FCs across functional networks. Critical FCs were identified based on their significantly greater contribution to higher ISFC in the AMC group relative to the control group. The thickness of lines within and between networks reflects the number of connections between nodes.

To further investigate group differences in inter‐individual similarity in the connectivity architectures of fronto‐parietal regions, an assessment of the inter‐individual similarity of functional connectivity (ISFC) was performed for each individual (see Experimental Section). A significantly higher ISFC was discernible in the training group relative to the control group (*t*(57) = 2.713, *p* = 0.004, Cohen's *d* = 0.698), indicating that AMC trainees exhibited more analogous FC patterns across individuals (**Figure** **4**A). The FCs that demonstrated a significantly higher contribution to the higher ISFC (*p* < 0.001) of the AMC group in comparison to the control group were identified as critical FCs (see Experimental Section). They were primarily located between the DAN and FPN and DMN (Figure 4B). Additionally, as anticipated, the individual's ISFC exhibited substantial congruence with individual‐to‐group similarity metrics (*r* = 0.99, *p* < 0.001; Figure S4, Supporting Information), validating ISFC as a reliable indicator of individualized connectivity architecture alignment with group‐level connectivity patterns.

Moreover, these functional connections, characterized by significant group differences, were pivotal in driving the greater ISFC observed as a result of the training. Notably, nearly 70% of these critical FCs exhibited significant group differences in connectivity values, with over 97% showing higher absolute FC values in the training group. This compelling evidence highlights the transformative impact of long‐term training on fronto‐parietal functional connections, revealing pronounced group differences not only in connectivity value but also in the individual variability of the connectivity architecture.

### 2.5 Relationship between ISFC and Behavioral Scores

**Figure 5:**
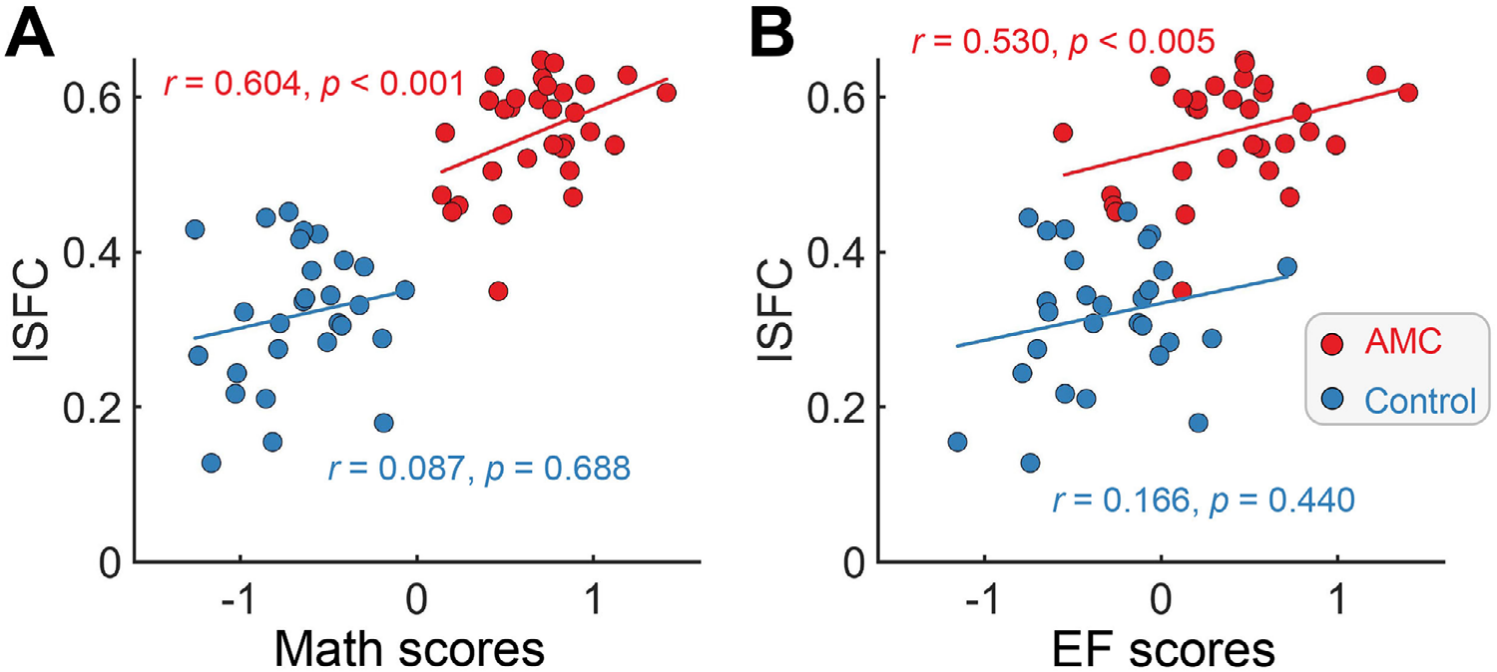
Brain‐behavior relationship using ISFC. (A) The ISFC showed significant correlations with mathematical ability scores (Math scores) and EF in the training group (*n* = 31, partial correlation, all *p* < 0.005), (B) but not in the control group (*n* = 28, partial correlation, all *p* > 0.05).

Given that an individual's ISFC serves as an indicator of their similarity to group‐level connectivity architecture, we expected that it might be associated with cognitive ability. The correlations between ISFC and EF and mathematical scores in each group were calculated by controlling for potential confounding variables, including age, sex, intelligence quotient (IQ), and head motion, using partial correlation. In the AMC group, we observed a significant positive correlation between the ISFC (dominated by critical DAN‐DMN‐FPN connections) and mathematical ability (*r* = 0.604, *p* < 0.001) as well as EF (*r* = 0.530, *p* = 0.005; **Figure** **5**). However, these relationships were not evident in the control group (mathematics: *r* = 0.087, *p* = 0.688; EF: *r* = 0.166, *p* = 0.440). The results remain significant after multiple comparison correction.

## 3 Discussion

The aim of the present study was to investigate the impact of long‐term AMC training on the relationship between domain‐specific abilities, taking into account EF and mathematical ability, and inter‐individual differences in these cognitive domains, as well as their neural correlates. The following key findings can be derived from the study, which utilized a dataset comprising a five‐year period of AMC training. At the behavioral level, individuals in the AMC training group demonstrated a significantly stronger correlation between EF and mathematical ability beyond their higher performance, accompanied by notably lower variability across individuals in these cognitive abilities. At the neural level, the training group displayed greater homogeneity in both inter‐domain networks and inter‐individual connectivity architecture when compared to the control group. Furthermore, the level of similarity in connectivity architecture to other individuals in the group was positively correlated with both EF and mathematical ability scores across the AMC‐trained children, unlike their peers in the control group. These findings offer valuable insights into the extensive impact of prolonged, multifaceted cognitive training, highlighting how such training can facilitate the integration of diverse cognitive abilities during development and shape their neural representation.

### 3.1 Dedifferentiation between EF and Mathematical Ability with Training

We found a stronger association between mathematical ability and EF in the training group relative to their peers, suggesting a stronger dependency of mathematical ability on EF. This might be supported by the distinctive strategy transition in mental arithmetic due to AMC training that progresses from a language‐based format to a visuospatial format.^[^ ^28^ ^]^ This suggests that when solving mental arithmetic problems with virtual beads, a greater reliance is placed on visuospatial processing of executive control than in language‐based digital number formats. A previous study also showed that prolonged abacus training strengthened the relationship between task switching and arithmetic/visuospatial abilities.^[^ ^52^ ^]^ Our findings extend this study to the broader view of EF and mathematical ability, suggesting an effect of visuospatial strategy on mental arithmetic and its cognitive structural organization.

Moreover, a stepwise multigroup analysis revealed that the inter‐individual variability of mathematical ability was lower in the training group. This observation is in alignment with our findings regarding arithmetic scores.^[^ ^53^ ^]^ To perform at a high expertise level in AMC, it is essential to adhere to the established rules governing the resolution of mental arithmetic with virtual beads. The homogeneity of cognitive strategy facilitates the uniform and efficient cognitive processing, which may be reflected in the inter‐individual differences in both cognition and brain function.^[^ ^46^ ^]^ Consequently, these inter‐individual differences are influenced by the cumulative amount of deliberate practice,^[^ ^40^, ^41^ ^]^ which shapes the cognitive architecture and fosters the development of specialized neural representations for enhanced neural efficiency.^[^ ^34^, ^54^ ^]^

The current investigation embarked on a comprehensive exploration of the multifaceted effects of abacus training, focusing specifically on its impact on the interrelationships and individual differences in EF and mathematical proficiency. While the SEM model shows acceptable fit indices (CFI = 0.938, SRMR = 0.082), the RMSEA (0.134) exceeds conventional thresholds. The fact that the RMSEA is larger than the threshold could be partly explained by the moderate sample size (*N* = 141), as RMSEA is known to be sensitive in smaller samples^[^ ^55^ ^]^ and has a low number of degrees of freedom. In such cases, the literature^[^ ^55^ ^]^ suggests prioritizing CFI and SRMR for model evaluation, especially given that factor loadings are satisfactory. Collectively, it is plausible to conclude that a multi‐domain cognitive training approach, employing a strategically tailored method across various cognitive domains, holds significant promise for fostering more robust interconnections between these cognitive systems. Furthermore, the uniformity and coherence of the cognitive strategy used can substantially influence the degree of individual differences observed after prolonged training. The present study underscores the effectiveness of a distinctive cognitive training approach in enhancing multiple cognitive trait domains, providing compelling evidence for the influential role of cognitive processes in shaping individual cognitive traits.

### 3.2 Neural Correlates of Training‐Induced Cognitive Dedifferentiation

To elucidate the training‐induced effects, we examined how the intervention modulated FCs, particularly within fronto‐parietal regions. The results demonstrated a distinct pattern: the AMC training group exhibited stronger FC between DAN, VAN, and FPN, alongside weaker FC between DMN and DAN. Among these networks, the DAN and VAN are crucial for directing and fine‐tuning attention^[^ ^56^, ^57^ ^]^ while the FPN is essential for cognitive control and task management.^[^ ^58^ ^]^ In contrast, the DMN facilitates introspection and self‐referential thinking by integrating information across the brain's cortical hierarchy.^[^ ^59^, ^60^ ^]^ Given the functional roles of these networks, the stronger connectivity between the DAN, VAN, and FPN in the training group suggests that AMC‐trained brains became more efficient at managing attentional control and cognitive demands during tasks. Conversely, the reduced coupling between the DAN and DMN signifies a transition towards more automatic and streamlined processing, with reduced reliance on deliberate, top‐down control. The present findings are consistent with those of previous research^[^ ^54^, ^61^ ^]^ and provide further evidence for the view that the brain becomes more specialized and efficient as it reallocates resources for improved task performance.

Corresponding to the stronger relationship between EF and mathematical ability within the training group, we observed greater overlap in the connectivity patterns linking these two cognitive domains. This neural evidence corroborates the stronger dependence of mathematical ability on EF, as discussed in existing literature on the increased recruitment of shared fronto‐parietal systems (such as posterior superior parietal lobe, PSPL) that support both cognitive domains through AMC training.^[^ ^32^, ^62^ ^]^ These findings indicate a strategic reorganization in mental arithmetic processing^[^ ^28^ ^]^ that may support the development of shared cognitive strategies (e.g., visuospatial manipulation), potentially benefiting both EF and mathematical abilities and enabling transfer effects between these domains. Notably, this dynamic stands in contrast to the documented neural competition observed between reading acquisition and face processing during developmental stages.^[^ ^63^ ^]^ Therefore, these findings resonate with the framework of training transfer,^[^ ^64^, ^65^ ^]^ which posits that cognitive training can enhance performance across tasks that share overlapping neural substrates, thereby underscoring the transformative potential of targeted interventions in shaping cognitive structures.

Moreover, the lower inter‐individual variability in latent abilities observed within the AMC training group was also reflected at the neural level, where greater ISFC within the fronto‐parietal regions was evident. According to the theory of deliberate practice,^[^ ^40^ ^]^ prolonged and focused training, such as that employed in abacus use, fosters greater neural homogeneity due to the adoption of a shared, consistent visuospatial strategy. In contrast, individuals in the control group, who employed traditional language‐based mental arithmetic strategies, exhibited more variable neural activation patterns, leading to higher inter‐individual variability in their neural profiles.^[^ ^46^ ^]^ These results align with our earlier observations, which demonstrated that long‐term abacus training promotes greater inter‐individual homogeneity in both mathematical traits and neural patterns across the brain.^[^ ^53^ ^]^

This neural evidence in the fronto‐parietal regions points to the network reconfigurations that may underlie the training‐induced changes in behavioral performance. It extends previous research by investigating the impact of training‐induced modulation on the connectivity architecture and converging FC value, inter‐domain similarity, and inter‐individual similarity in brain connectivity patterns. By doing so, it sheds light on the dedifferentiation of neural correlates that may arise from the adoption of a unique visuospatial strategy, offering a novel perspective on how specific cognitive training interventions can reshape the neural representations of both mathematical and EF abilities.

### 3.3 Averaged Inter‐Individual Similarity of Connectivity Architecture is Associated with Cognitive Ability

Given the strong consistency between ISFC and individual‐to‐group similarity in connectivity architecture, the present study demonstrated a positive correlation between ISFC and both EF and mathematical performance scores across individuals in the training group. This finding aligns with the notion that the shared memory content of cognitive strategies, shaped by the accumulation of cognitive skills with practice,^[^ ^50^, ^51^ ^]^ is associated with cognitive ability—here represented by EF and mathematical ability scores—via individual‐to‐group similarity in connectivity architecture. Previous studies have also suggested that individual‐to‐group similarity in brain activity or connectivity may reflect the extent to which each student has mastered a specific skill during learning.^[^ ^66^, ^67^ ^]^ Prolonged deliberate practice, rooted in shared experiences and memory, is thought to shape brain connectivity architecture toward a shared structure in neural representation across individuals.^[^ ^40^, ^41^, ^51^ ^]^ This provides novel evidence of shared memory in the learning process, as reflected in changes in both neural activity and cognitive ability.

### 3.4 Limitations

Whilst the present findings provide valuable first evidence into the neural correlates underlying training‐induced cognitive changes, it is important to consider the methodological limitations of the study. First, although the experiment utilized a longitudinal design, the emphasis was primarily placed on post‐training assessments following a five‐year training period, owing to the unavailability of pre‐training behavioral assessments on mathematical ability. Nevertheless, group matching based on demographic and cognitive variables helped to mitigate potential baseline differences between groups (see Supporting Information). Second, while conducting cognitive assessments in school helped to retain most participants throughout the longitudinal study, a high attrition rate was experienced during brain imaging sessions conducted outside of school. Notwithstanding the inherent challenges in longitudinal research, our current findings establish a robust foundation for future investigations on larger cohorts and more comprehensive assessments. Third, the current study did not incorporate task‐based fMRI data, but emphasizes the critical need for future research to implement multi‐modal neuroimaging approaches. Such comprehensive methodological integration would facilitate a more nuanced understanding of how AMC training modulates functional brain networks. Furthermore, it would enable the precise identification of neural signatures associated with training‐induced cognitive enhancement.

## 4 Conclusion

The present study provides compelling evidence for the remarkable plasticity of cognitive architecture, demonstrating how sustained training induces substantial dedifferentiation across multiple dimensions. These include the relationships between EF and mathematical competencies, inter‐individual variability of these cognitive domains, and their corresponding neural substrates within fronto‐parietal circuits. Of particular significance is the systematic acquisition of a highly structured cognitive strategy through AMC training, which facilitates a fundamental shift toward visuospatial processing modalities and promotes a more unified approach to arithmetic operations. This strategic reorganization manifests in stronger integration between EF and mathematical ability, accompanied by lower domain difference. These cognitive modifications are notably reflected in increased pattern similarity, both in terms of inter‐domain neural network organization and inter‐individual connectivity architectures. The observed multifaceted effects on EF and mathematical ability suggest broader implications for skill transferability, underscoring the potentially transformative role of multi‐domain cognitive training interventions in reshaping cognitive structures. These findings not only advance our theoretical understanding of cognitive plasticity but also offer valuable insights for evidence‐based educational practices and interventions.

## 5 Experimental Section

### Participants

In the current study, a total of *n* = 142 children were enrolled, comprising a training group of *n* = 72 participants and a control group of *n* = 70 participants, as part of the Chinese Abacus Training Project (CATP). This longitudinal dataset was designed to investigate the effects of prolonged abacus training on brain and behavior (refer to Supporting Information for comprehensive details). The experimental condition involved the training group receiving 2 h of AMC instruction per week over a five‐year period, commencing in first grade and concluding at the onset of sixth grade. Conversely, the control group, which had no prior exposure to abacus techniques, received supplementary instruction in conventional academic subjects, including mathematics and reading. The current investigation focused on the post‐training phase to elucidate the impact of AMC training on the research question at hand. At this juncture, all participants except one had completed EF and mathematics‐related behavioral assessments, with the groups carefully matched for age, sex, and IQ (refer to Table S1, Supporting Information for detailed demographic information). A subset of 66 children (training group: *n* = 34; control group: *n* = 32) underwent resting‐state fMRI scans. Following the exclusion of seven participants due to excessive head motion (defined as displacement exceeding 3.0 mm or rotation surpassing 3 degrees) or suboptimal imaging quality, the final fMRI sample comprised 59 children. The final sample was comprised of 31 participants from the training group (*M*_age_ = 11.94 ± 0.52 years, 15 females) and 28 participants from the control group (*M*_age_ = 12.09 ± 0.51 years, 13 females). These data were subsequently subjected to further analysis to address the research objectives.

### Data Acquisition and Preprocessing

Magnetic resonance imaging (MRI) was performed utilizing a 1.5 T Philips MRI scanner (Achieva, Philips), equipped with an eight‐channel head coil to enhance signal reception. The resting‐state fMRI scan was conducted over a duration of 360 s, yielding a total of 180 volumes through a single‐shot echo planar imaging (EPI) sequence. The specific parameters for this EPI sequence were established as follows: repetition time (TR) = 2000 ms, echo time (TE) = 50 ms, flip angle (FA) = 90 degrees, slice thickness/gap = 5 mm/0.8 mm, field of view (FOV) = 230 × 230 mm^2^, matrix size = 64 × 64, and 22 interleaved ascending slices per volume. To obtain high‐resolution structural images, a 3D fast field echo (FFE) sequence was employed, with parameters set at TR = 25 ms, TE = 4.6 ms, FA = 15 degrees, FOV = 256 × 256 mm^2^, acquisition matrix = 256 × 256, voxel size of 1 × 1 × 1 mm^3^, and a total of 150 slices acquired in the sagittal plane.

The preprocessing of resting‐state fMRI data was conducted using the Data Processing Assistant for Resting‐state fMRI Advanced Edition (DPARSF‐A, version 4.2) toolbox.^[^ ^68^ ^]^ Initially, the first five volumes were discarded to allow for signal equilibrium, after which the remaining 175 volumes underwent slice timing correction. The imaging data were subsequently realigned to correct for inter‐TR head motion. A multiple linear regression analysis was then applied to eliminate the effects of several nuisance variables, including the average signals from white matter, cerebrospinal fluid (CSF), global signals, and the 24 Friston body motion parameters. The functional images were normalized to the Montreal Neurological Institute (MNI) space, with a resampled voxel size of 4 × 4 × 4 mm^3^. Following normalization, the functional images were smoothed using a 6 mm full‐width half‐maximum (FWHM) Gaussian kernel, subjected to linear detrending, and filtered with a bandpass filter set to 0.01–0.1 Hz. Finally, time series for each region of interest (ROI) were generated for subsequent analysis, thereby facilitating a comprehensive evaluation of the resting‐state functional connectivity.

### Cognitive Measures

The cognitive assessment battery employed in this investigation comprised five tasks: two specifically designed to evaluate mathematical competencies and three to assess EF abilities. The mathematical evaluation comprised two distinct components: a comprehensive arithmetic assessment and a sophisticated mathematics‐related visuospatial examination. These were complemented by the Compare‐to‐5 task, which was specifically engineered to evaluate numerical magnitude processing capabilities. The EF assessment protocol incorporated three well‐validated paradigms: the N‐back task for working memory evaluation, the Go/No‐go task for inhibitory control assessment, and the Dots task for measuring cognitive flexibility.

### Definition of the Regions of Interest (ROI)

To systematically identify brain regions of interest (ROIs) associated with EF and mathematical processing, meta‐analytic activation maps derived from Neurosynth were used.^[^ ^69^ ^]^ The analytic pipeline comprised three primary steps: Initially, a comprehensive meta‐analytic map was generated by integrating item‐based meta‐analytic maps corresponding to core cognitive components (i.e., “inhibition”, “shifting”, “working memory,” and “arithmetic”). This process was undertaken in order to retain only voxels that exceeded an adapted Z‐score threshold of 3.^[^ ^70^ ^]^ Second, the combined activation map was projected onto the Schaefer functional atlas, which is comprised of 400 parcellations distributed across seven canonical brain networks. The spatial overlap was then quantified using a filled degree metric, which was defined as the proportion of meta‐analytically activated voxels within each atlas‐defined ROI. ROIs exhibiting robust functional relevance (filled degree >40%) were retained, yielding 92 regions for subsequent analysis (Figure S5, Supporting Information). Finally, region‐to‐region FC matrices were constructed by computing Pearson correlations between the resting‐state BOLD time series of all possible ROI pairs. This provided a comprehensive characterization of the functional architecture underlying EF and mathematical processing.

### Characterization of Behavior‐Associated FC Patterns

To elucidate the neural convergence of functional circuits underlying EF and mathematical ability in AMC‐trained versus control cohorts, a systematic analytical framework was implemented in order to identify behavior‐associated FC patterns. The methodological approach comprised three primary components:

1. Estimation of Latent Cognitive Ability Scores Using Structural Equation Modeling (SEM): Confirmatory SEM was employed to derive latent variables representing core cognitive constructs. The mathematical ability factor was extracted from shared variance across arithmetic operations, visuospatial processing, and numerical processing tasks. Similarly, the EF factor captured common variance among inhibitory control, cognitive flexibility (shifting), and working memory assessments. Individual factor scores were computed to obtain estimates of latent cognitive abilities.
2. Robust Identification of Behavior‐Associated FC: In order to guarantee methodological robustness in the identification of behavior‐relevant neural patterns, a leave‐one‐subject‐out cross‐validation approach was implemented for each cohort. The following steps were taken for each iteration: Training sets (*n*‐1 subjects) were generated, comprising FC matrices and corresponding behavioral metrics. Partial correlations were computed between individual FCs and behavioral measures, controlling for demographic variables (age, sex), general cognitive ability (IQ), and head motion parameters. Finally, significantly correlated connections (*p* < 0.01) were identified to construct binary, unweighted behavior‐associated connectivity patterns. The results from each iteration were then aggregated to derive stable, representative connectivity patterns for each cognitive domain.
3. Quantification of Network Similarity between Cognitive Domains: To assess the degree of neural overlap between EF and mathematical processing networks, the normalized angle similarity between their respective connectivity patterns was computed:
(1) $$patternsimilarity\left(A,B\right)=1-\mathrm{acos}\left(\frac{A\cdot B}{\left|A\right|\left|B\right|}\right)/pi$$

where *A* and *B* represent vectorized upper‐triangular connectivity matrices corresponding to EF and mathematical ability patterns, respectively. This metric provides a normalized [0,1] index of pattern convergence, with higher values indicating greater neural overlap between cognitive domains.

### Inter‐Individual Similarity of Functional Connectivity (ISFC)

A higher ISFC value for an individual was indicative of a greater degree of similarity between that individual's FC profile and the FC profiles of all other participants in the study.^[^ ^47^, ^48^, ^49^ ^]^ In the present investigation, the ISFC for each individual was estimated through the following methodical steps:

1. For each participant, an FC matrix was constructed by calculating the Pearson correlations of time series within selected ROIs, yielding a 92 × 92 correlation matrix. In the ensuing stage of the process, the individual FC matrix was vectorized by the extraction of the upper triangular elements, with the diagonal elements being excluded in order to eliminate redundancy. This process resulted in a vector comprising 4186 elements.
2. The homogeneity matrix for all participants within each group was assessed by computing Pearson correlations between the vectorized FC profiles of individuals, thereby producing an *n* × *n* similarity matrix (where *n* denotes the number of participants in each group).
3. The individual ISFC was determined by averaging the values of each row in the homogeneity matrix, which served as the ISFC profile for that participant. The individual ISFC for each participant can be mathematically expressed as follows:
(2) $$ISFC_{i}=\frac{1}{n-1}\sum_{\begin{array}{c} j=1\\ j\neq i \end{array}}^{n}Corr\left(X_{i},X_{j}\right)$$

where *X_i_* and *X_j_* represent the vectorized FC profiles of participant *i* and participant *j*, respectively.

### Critical Functional Connectivity Analysis of ISFC

The principal functional networks responsible for the observed ISFC variations were systematically identified by conducting a comprehensive critical FC assessment. The analytical process was structured as follows:

1. FC‐wise loading determination: Utilizing the vectorized individual FC matrices (*n* × 4186) derived from the previous ISFC calculation, the FC‐wise loadings for each participant were quantified through a rigorous normalization and averaging procedure. Specifically, the element‐wise product of normalized FC profiles across participants within each group was computed. The *k*‐th FC‐wise loading for an individual was thus mathematically formulated as:
(3) $$Loadings_{i}\left(k\right)=\frac{1}{n-1}\;\sum_{\begin{array}{c} j=1\\ j\neq i \end{array}}^{n}Z_{i,k}*Z_{j,k}$$

This procedure yielded a comprehensive *n* × 4186 FC‐wise loading matrix encompassing all participants.

2) Critical FC identification and network mapping: A systematic identification of FCs was performed, identifying statistically significant differential contributions between the AMC group and the control group using a threshold of *p* < 0.001. These differentially modulated connections, termed “critical FCs,” were characterized through network‐level mapping and quantitative analysis. By precisely localizing critical FCs within functional networks and calculating their proportional distribution across between‐network and within‐network connectivity over the total number of critical FCs, a nuanced assessment of brain network topological reorganization was conducted.

### Statistical Analyses—Multiple Group Analysis

To investigate potential group differences in the relationship between EF and mathematical ability, a stepwise analysis of behavioral data (*n* = 141) was conducted utilizing multi‐group SEM via the lavaan package in R software.^[^ ^71^ ^]^ As demonstrated in the Figure S6 (Supporting Information), the latent construct of mathematical ability was operationalized through the following four observed variables: arithmetic ability and mathematics‐related visuospatial ability, derived from mathematics assessments, alongside numerical magnitude processing ability, which was evaluated through response times (RTs) under two conditions (proximal and distal) in the Compare‐to‐5 task. The latent variable of EF was estimated using three observed performance metrics: working memory, assessed via the N‐back task; inhibition, measured by the Go/No‐go task; and shifting ability, evaluated through the Dots task. A series of stepwise model comparison tests was employed to discern group differences between the AMC and control groups, adhering to a standardized sequence of measurement invariance assessments.^[^ ^72^ ^]^

1. In Model 0, the factorial structure of EF and mathematical ability, as well as their interrelationship, were evaluated using the behavioral scores from all participants.
2. In Model 1, the factorial structure established in Model 0 was fitted to both the training and control groups in order to test for configural invariance. This allowed all model parameters, including the correlation between EF and mathematical ability as latent constructs, to be freely estimated.
3. Model 2 imposed constraints on factor loadings, requiring them to be equal across both groups in order to assess loading invariance (also referred to as weak invariance).
4. In Model 3, further constraints were placed on both the factor loadings and variances of the latent variables, necessitating equality across groups.
5. Model 4 was built upon this framework by constraining the co‐variances of the latent variables to be equal across groups, alongside the established equivalence of factor loadings and variances within both groups.

Subsequent model comparison tests were executed between successive models (Model 1 vs Model 2, Model 2 vs Model 3, Model 3 vs Model 4) employing the χ^2^ difference test. This systematic approach enabled the examination of group differences in model parameters. For further analyses, latent scores for EF and mathematical ability were extracted from Model 2, yielding estimates of these two constructs for subsequent associations with neural measures.

### Statistical Analyses—Permutation Test for Between‐Group FC Differences

To systematically evaluate FC differences between the AMC and control groups, a permutation test (10 000 iterations) was conducted to correct for multiple comparisons. In each iteration, group labels of individuals were randomly shuffled, and candidate edges showing potential differences were identified using independent samples *t*‐tests (primary threshold at uncorrected *p* < 0.05). The differential connections were divided into two categories: In the AMC group, Category 1 demonstrated stronger connectivity in comparison to the control group, while Category 2 exhibited weaker connectivity. This enabled the assessment of the network distribution of candidate differential FC,^[^ ^73^ ^]^ quantified as the proportion of group‐differentiated connections within and between functional networks (normalized to the total number of differential connections). The permutation procedure generated an empirical null distribution, thereby enabling statistical comparison of observed network‐level effects. Corrected *p*‐values were derived by comparing the permuted proportion of significant connections to the actual data, applying a 5% significance threshold after correction. This approach unveils training‐induced changes in functional architecture, highlighting network reorganization linked to AMC expertise. Besides, given an fMRI sample size of 60, the group‐level comparison analysis of brain indicators (e.g., FC or ISFC) estimated by independent samples *t*‐test achieves 76% statistical power to detect a target effect size of 0.7 (Cohen's *d*) at an alpha level of 0.05, and a power of 86% to detect an effect of 0.8.

### Statistical Analyses—Correlation Analysis of Brain‐Behavior Relationships

The relationship between ISFC and cognitive performance (EF and mathematical ability) was investigated using correlational analyses. Cognitive metrics were derived as latent variables via SEM using lavPredict package in R,^[^ ^74^ ^]^ ensuring theoretically clean constructs and minimizing measurement error. The ISFC of each participant was evaluated, with a primary focus on three key networks—the DAN, DMN, and FPN—due to their prominent contributions to higher ISFC in the AMC group. Partial correlation was applied to quantify brain‐behavior relationships while controlling for age, sex, IQ, and head motion, isolating specific neural‐cognitive associations.

## Conflict of Interest

The authors declare no conflict of interest.

## Supporting information



Supporting Information

## Data Availability

The data that support the findings of this study are available from the corresponding author upon reasonable request.
