# Supplementary material for: Dedifferentiation and Transfer in Executive Function and Math Ability Following a Five‐Year Abacus Training in Schoolchildren
Source: Adv Sci (Weinh). 2025 Jul 11;12(37):e04518. doi: 10.1002/advs.202504518 (PMC12499462; doi:10.1002/advs.202504518)
Supplement: Supplementary file 1 — Supporting Information [file ADVS-12-e04518-s001.docx]

Supporting Information

Dedifferentiation and Transfer in Executive Function and Math Ability Following a Five-Year Abacus Training in Schoolchildren

Tianyong Xu ^#^, Xinyang Liu^#^, Hongjian He, Changsong Zhou*, Andrea Hildebrandt*, Feiyan Chen*

Main Content

- Supplementary Methods
- Supplementary Figures
- Supplementary Tables

**Supplementary Methods**

*Chinese Abacus Training Project (CATP)*

The Chinese Abacus Training Project (CATP) was a five-year longitudinal study designed to examine the brain and behavioral changes resulting from long-term abacus-based mental calculation (AMC) training. As a core component of this longitudinal investigation, the initial recruited cohort comprised 186 school-age children (aged 6-8 years) who were beginning their first-grade education in China, following the exclusion of participants with incomplete demographic profiles. All participants were enrolled in standardized primary school curricula. Given the random allocation within the Chinese primary education system, students from those pre-formed classes were assigned to either the AMC intervention group (*n* = 87, including 48 females) or the control condition (*n* = 99, including 48 females). This allocation methodology ensured matching across key demographic and cognitive variables including age, sex, intelligence quotient (IQ), early school behavior rating scale scores, and mastery motivation indices (detailed in Table S2). Cognitive abilities and neuroimaging data were assessed with one to four assessments conducted during the whole training period. This approach yielded a longitudinal dataset of prolonged training, thus facilitating comprehensive analysis of behavioral and neuroimaging changes over time. The protocol of the present study was reviewed and endorsed by the ethics research committee of Zhejiang University in China. The study was conducted in accordance with the approved guidelines and regulations. Informed consent was obtained from all participants and their parents.

*Training Procedure*

The participants in the experimental group underwent an intensive, longitudinal five-year AMC training program, with weekly sessions totaling two hours of specialized instruction. The curriculum has been meticulously designed to progress systematically through multiple stages of cognitive and computational development. Initially, subjects were introduced to the fundamental principles and mechanics of physical abacus manipulation, with a primary focus on mastering basic arithmetic operations: addition, subtraction, multiplication, and division. Upon demonstrating proficiency in these foundational skills, the training paradigm transitioned to mental calculation techniques, wherein participants were required to visualize and manipulate virtual abacus beads through cognitive processes alone. During the preliminary stages of this training regimen, kinesthetic aids in the form of finger movements were actively encouraged to facilitate the internalization of the abacus framework. As participants' proficiency increased, there was a gradual shift towards discouraging reliance on these physical cues, with a heightened emphasis on enhancing both the speed and accuracy of purely mental computations. This protracted training period facilitated the development of highly refined AMC skills, enabling participants to tackle progressively complex arithmetic problems as the difficulty gradient of their practice steadily increased. In contrast, the control group received no formal instruction in either physical or mental abacus techniques. These participants instead adhered to a traditional school-based curriculum, devoid of exposure to the specialized AMC training provided to their counterparts in the experimental group.

*Efficacy of Abacus Training*

The efficacy of AMC training has been demonstrated by the finding that, upon completion of the training program, the majority of participants in the training group demonstrated a high level of proficiency in AMC skills. This was evidenced by their performance on the Standard Mental Abacus Level Test (SMALT, Table S3). This assessment evaluates mental abacus proficiency among children in the training group, with Level 1 representing the highest achievable performance tier. Specifically, post-training assessments of SMALT revealed that 93% of trainees who completed the evaluation achieved high proficiency, with 87% attaining Level 1 and an additional 4% reaching Level 2. In contrast, the control group did not demonstrate comparable levels of skills, thereby emphasizing the efficacy of the training program.

*Cognitive Assessment Battery—Mathematical Ability Assessment*

Mathematical competencies were systematically assessed using the Heidelberg Rechentest (HRT), a validated paper-and-pencil assessment instrument that has demonstrated robust psychometric properties, including high reliability coefficients within the Chinese population. ^[1,2]^ The HRT consists of two distinct subscales designed to measure discrete components of mathematical cognition: arithmetic ability and visuospatial mathematical processing. The arithmetic subscale includes six timed subtests that assess basic mathematical operations and numerical reasoning: addition, subtraction, multiplication, division, equation completion and numerical comparison tasks. The Visuospatial subscale includes five timed subtests that assess spatial-numerical processing: line estimation, pictorial enumeration, three-dimensional cube counting, sequential number counting, and numerical pattern sequencing. To optimize test administration and facilitate participants' familiarization with the assessment protocol, an initial number-copying subtest was administered as a pretest. Throughout the assessment, participants were required to provide written responses using standardized procedures within specified time limits. Individual performance measures were quantified by converting raw scores from each subtest into standardized T-scores based on established norms for the Chinese urban population. ^[2]^ Subsequently, composite scores for both arithmetic and visuospatial skills were then calculated by taking the mean T-scores across the respective subtests within each subscale, providing standardized measures of these different mathematical skills.

*Cognitive Assessment Battery—Compare-to-5 Task*

The experimental paradigm implemented a binary distance-based classification protocol, with systematic manipulation of numerical proximity to the reference digit '5'. The stimulus set was dichotomized into two distinct conditions based on numerical proximity: proximal digits (3, 4, 6, 7) and distal digits (1, 2, 8, 9). The experimental protocol comprised 96 discrete trials, with equivalent distribution across conditions (48 trials per condition). The temporal structure of each trial adhered to a standardized sequence: initiation with a 500ms fixation cross presentation, followed by the target numerical stimulus, and termination with a 500ms fixation cross. Participants were instructed to execute rapid, accurate binary classifications via designated response keys, indicating whether the presented digit was numerically greater or lesser than the reference value '5'. The target stimulus remained visible until response execution. To minimize potential sequential effects and response priming, stimulus presentation was randomized with the constraint that consecutive trials could not feature identical numerical values. Response latency was operationalized as the mean reaction time (RT) for accurate target classifications within each condition. This paradigm facilitates precise quantification of numerical magnitude processing efficiency under varying conditions of numerical distance, thereby providing a sensitive measure of fundamental numerical representation and comparison processes in both proximal and distal numerical contexts.

*Cognitive Assessment Battery—N-back Task*

Working memory capacity was evaluated using the N-back paradigm, a well-established cognitive assessment instrument that has demonstrated robust validity in measuring working memory processes. ^[3,4]^ The present investigation utilized a 3-back task, comprising 10 discrete experimental blocks, with each block consisting of 12 trials and a predetermined target frequency of three trials per block. The temporal and spatial parameters of stimulus presentation were precisely controlled: each trial presented a white square stimulus against a 3×3 black matrix grid for a duration of 500ms, followed by an interstimulus interval of 2500ms. The allocation of stimulus positions was determined by means of a pseudo-randomization algorithm, with the objective of ensuring appropriate distribution of target and non-target trials. The target stimuli were operationally defined as presentations matching the spatial location of the stimulus occurring three positions prior in the sequence (3-back condition), while all other presentations were classified as non-targets. Participants were instructed to execute motor responses exclusively for target stimuli. The performance of the participants was measured using signal detection theory, with the discrimination sensitivity index *d'* being calculated as the standardized difference between the hit rate and the false alarm rate (*d'* = *z*_target hit rate_ - *z*_false alarm rate_). This metric provides a bias-free measure of working memory performance by accounting for both accurate target detection and incorrect responses to non-targets.

*Cognitive Assessment Battery—Go/No-go Task*

The Go/No-go paradigm was implemented in order to evaluate response inhibition capabilities through a selective response protocol that utilized taxonomically distinct visual stimuli. Participants were instructed to execute motor responses to diverse animal stimuli (go trials) while selectively suppressing responses to presentations of chimpanzee stimuli (no-go trials), thereby establishing a prepotent response tendency that required active inhibition on no-go trials. The temporal parameters of stimulus presentation were meticulously regulated, with each visual stimulus displayed for a duration of 500ms, followed by an inter-stimulus interval ranging from 1100ms to 1200ms, with the objective of minimizing temporal expectancy effects. The experimental protocol commenced with a practice block comprising 12 trials to ensure task comprehension and response criterion acquisition, followed by two formal experimental blocks, each containing 70 discrete trials. The ratio of trial types was systematically manipulated, with no-go trials constituting 20% of the formal experimental trials, creating an asymmetric probability structure that established a strong prepotent response tendency. The inhibitory control performance of the participants was measured by the mean accuracy rates across both go and no-go conditions. This provided a composite measure of response execution and inhibition capabilities.

*Cognitive Assessment Battery—Dots Task*

A modified version of the Dots paradigm was implemented to evaluate cognitive flexibility capability. Each discrete trial adhered to a standardized temporal sequence: initial fixation presentation (500ms), followed by an inter-stimulus interval (500ms), target stimulus presentation (maximum 750ms, terminating upon response detection), and a post-response interval (500ms). The experimental protocol comprised three distinct blocks with systematically varied task demands. The congruent and incongruent blocks each contained 20 trials, while the mixed block incorporated 61 trials. Target stimuli consisted of either striped or grey dots (1cm diameter) presented laterally on the display. Response execution required bilateral manual responses via designated keys ('f' and 'j'). In the congruent block, participants responded to a single dot type following a spatial congruency rule (ipsilateral response). The incongruent block necessitated response inhibition of the previously established rule, requiring contralateral responses to the alternate dot type. The mixed block integrated both stimulus types and their associated response rules, demanding flexible rule switching (e.g., striped dots - ipsilateral response; grey dots - contralateral response). To control for potential stimulus-response mapping effects, rule assignment was counterbalanced across participants: half the sample responded ipsilaterally to striped dots and contralaterally to grey dots, while the remainder followed the inverse mapping. Task comprehension was ensured through four practice trials preceding each experimental block. Cognitive flexibility was quantified through the computation of switching costs within the mixed block, operationalized as the differential reaction time between switch and non-switch trials (RT_switch trials_ - RT_non-switch trials_).

References

1. J. Haffner, K. Baro, P. Parzer, F. Resch, H. Rechentest, Diagnostik Math Neue Folge Band 2005, 4, 125.

2. H. Wu, L. Li, Chinese Journal of Public Health 2005, 21, 473.

3. A. Hockey, G. Geffen, Intelligence 2004, 32, 591.

4. S. M. Jaeggi, B. Studer‐Luethi, M. Buschkuehl, Y.‐F. Su, J. Jonides, W. J. Perrig, Intelligence 2010, 38, 625.

**Supplementary Figures**


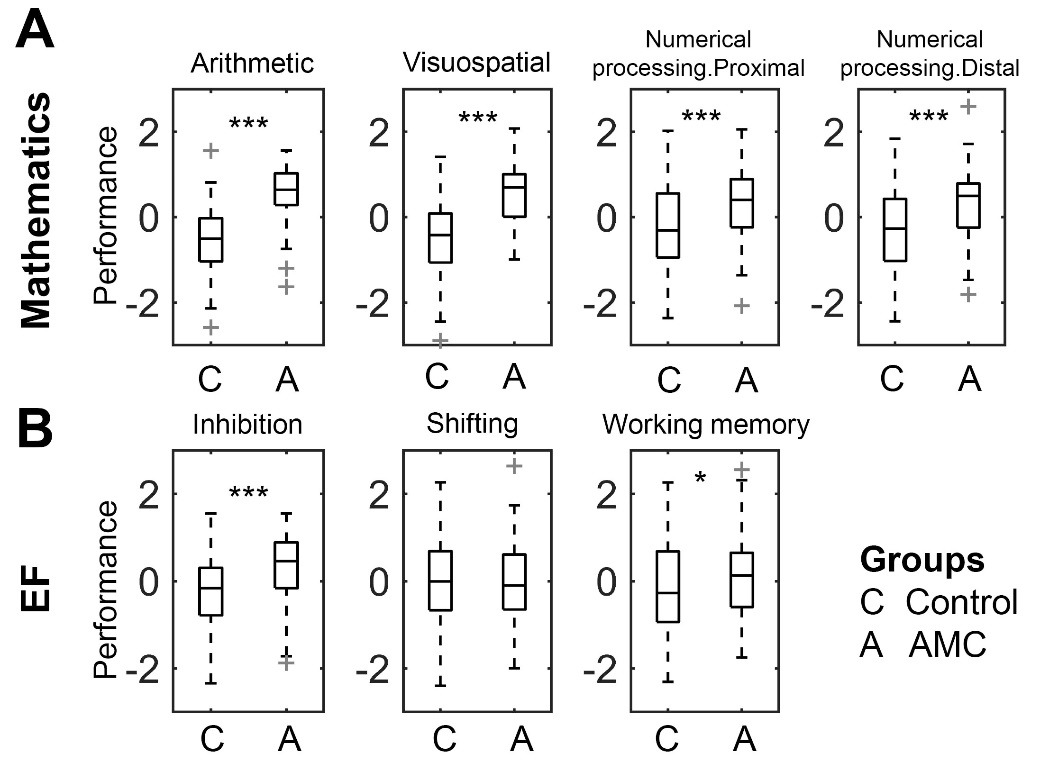


**Figure S1.** Behavioral performance in the mathematical subtests (top row) and the EF subtests (bottom row). All behavioral scores were standardized. A higher score is indicative of superior performance. The independent samples *t*-tests were employed to reveal significant group differences in cognitive abilities across multiple domains. Specifically, the training group demonstrated significantly superior arithmetic performance (*t*(125) = 8.661, *p* < 0.001, Cohen’s *d* = 1.537) and mathematical visuospatial ability (*t*(125) = 6.839, *p* < 0.001, Cohen’s *d* = 1.214) compared to the control group. Numerical magnitude processing was also significantly higher in both proximal (*t*(135) = 3.488, *p* < 0.001, Cohen’s *d* = 0.596) and distal conditions (*t*(135) = 3.702, *p* < 0.001, Cohen’s *d* = 0.633)) for the training group. Regarding EF abilities, significant differences were observed in inhibition (*t*(137) = 3.866, *p* < 0.001, Cohen’s *d* = 0.656) and working memory (*t*(134) = 2.140, *p* = 0.034, Cohen’s *d* = 0.367), but not shifting ability (*t*(130) = -0.787, *p* = 0.433, Cohen’s *d* = 0.137). Note: *** *p* < 0.001, ** *p* < 0.01, * *p* < 0.05.


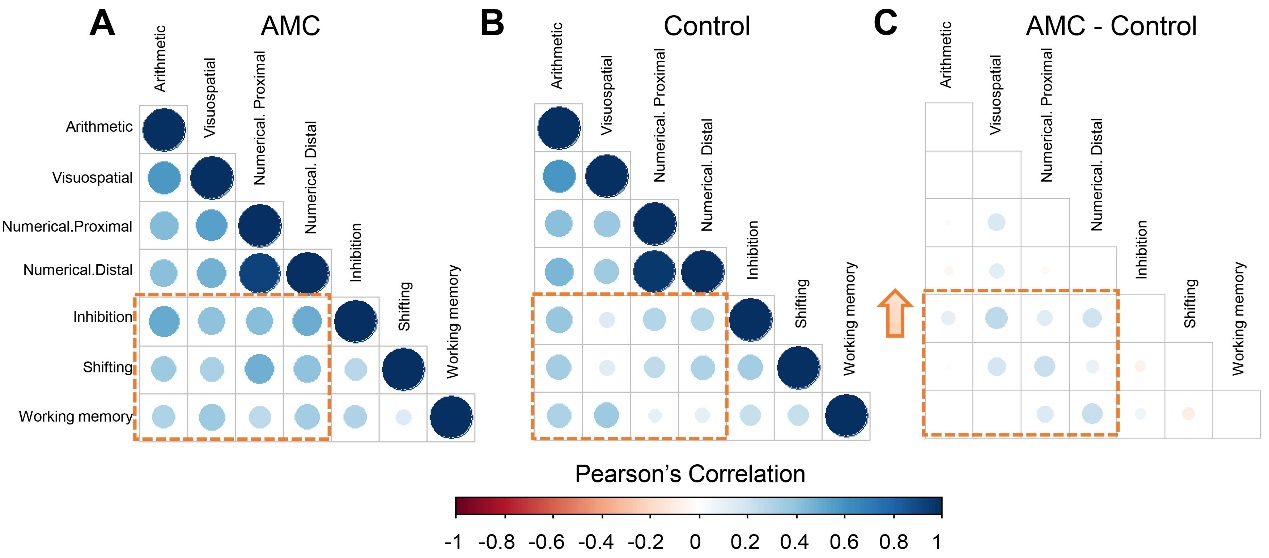


**Figure S2.** Correlation analysis at the level of single behavioral measurement within groups (A and B) and between groups (C). The training group (*n* = 31, Pearson’s correlation) showed higher correlations between EF and mathematical ability than the control group (*n* = 28, Pearson’s correlation).


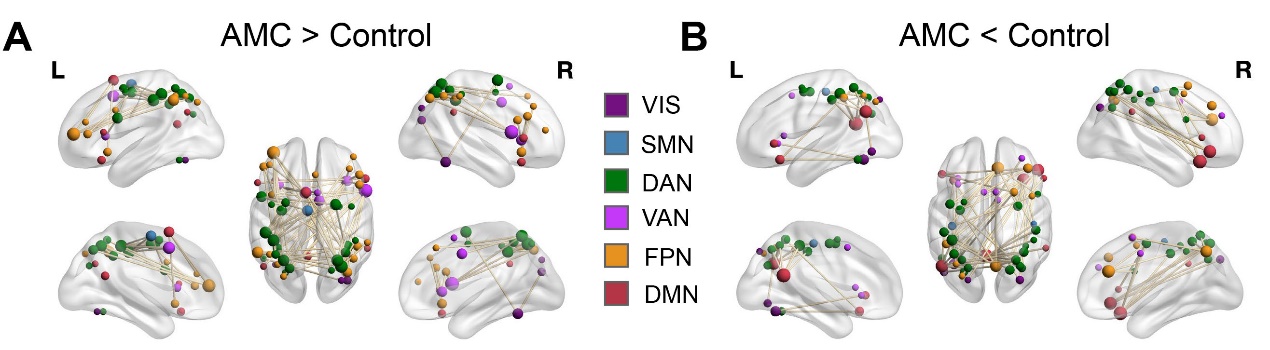


**Figure S3.** Topographical distribution of FCs with group difference. Node size represents node degree, defined as the total number of FCs connected to that node.


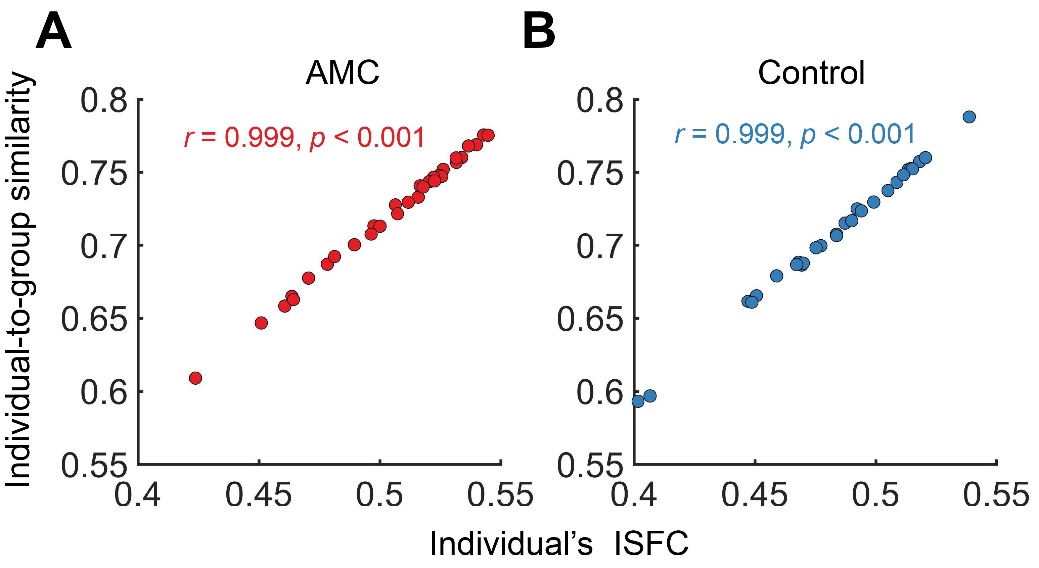


**Figure S4.** The ISFC of an individual reflects the similarity of their connectivity architecture to that of the group (training group: *n* = 31, Pearson’s correlation, *p* < 0.001; control group: *n* = 28, Pearson’s correlation, *p* < 0.001).


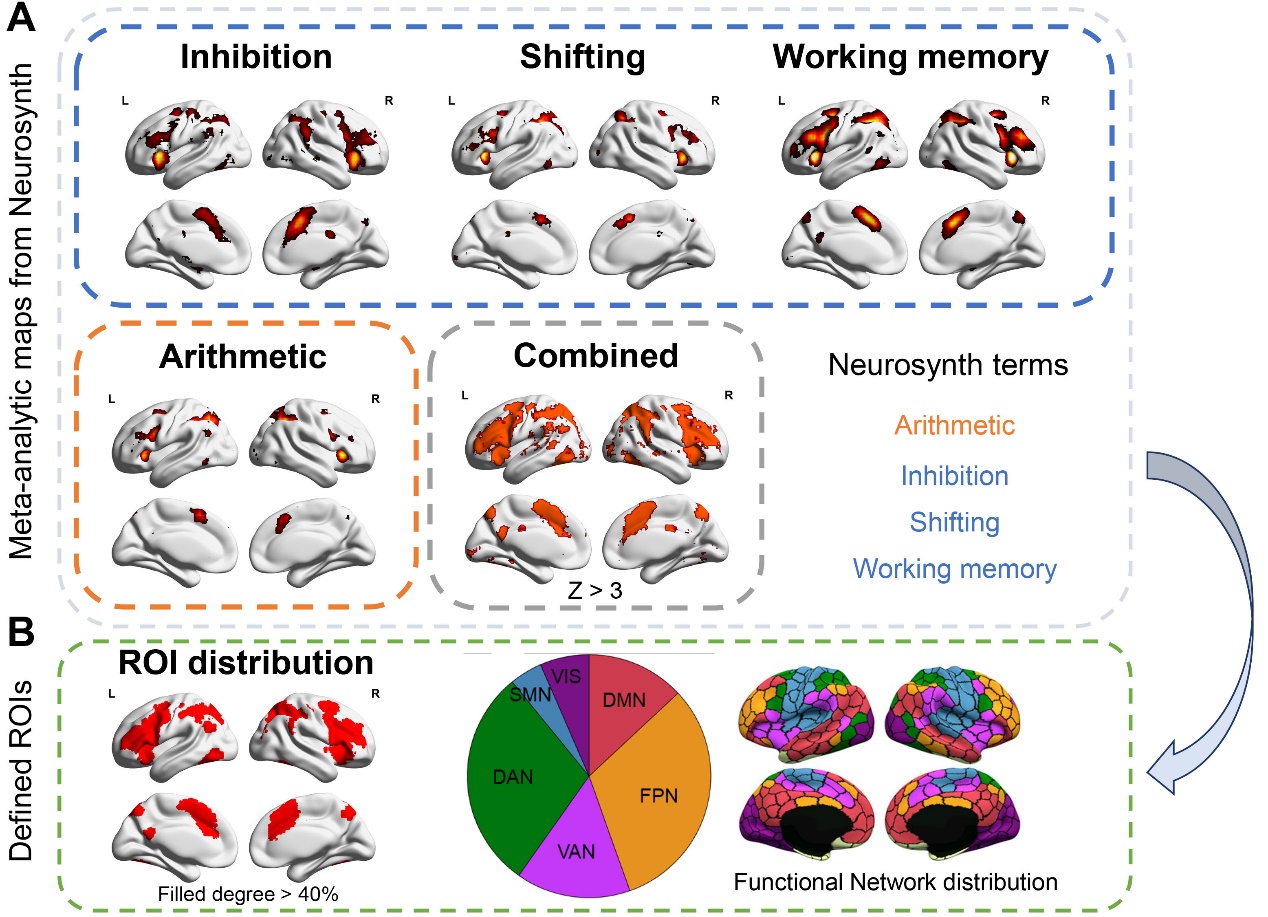


**Figure S5.** The predefined ROIs. (A) Meta-analytic activation maps for EF- and mathematics-related terms were extracted from Neurosynth and synthesized to generate a unified probabilistic map representing their joint neural substrates. (B) The spatial organization and network distribution of the final ROIs were visualized following quantitative filtration (40% threshold) using Schaefer's functional atlas, enabling precise characterization of the ROI-level organization. *Abbreviations*: VIS, visual network; SMN, sensory/motor network; DAN, dorsal attention network; VAN, ventral attention network; FPN, frontoparietal network; DMN, default mode network.


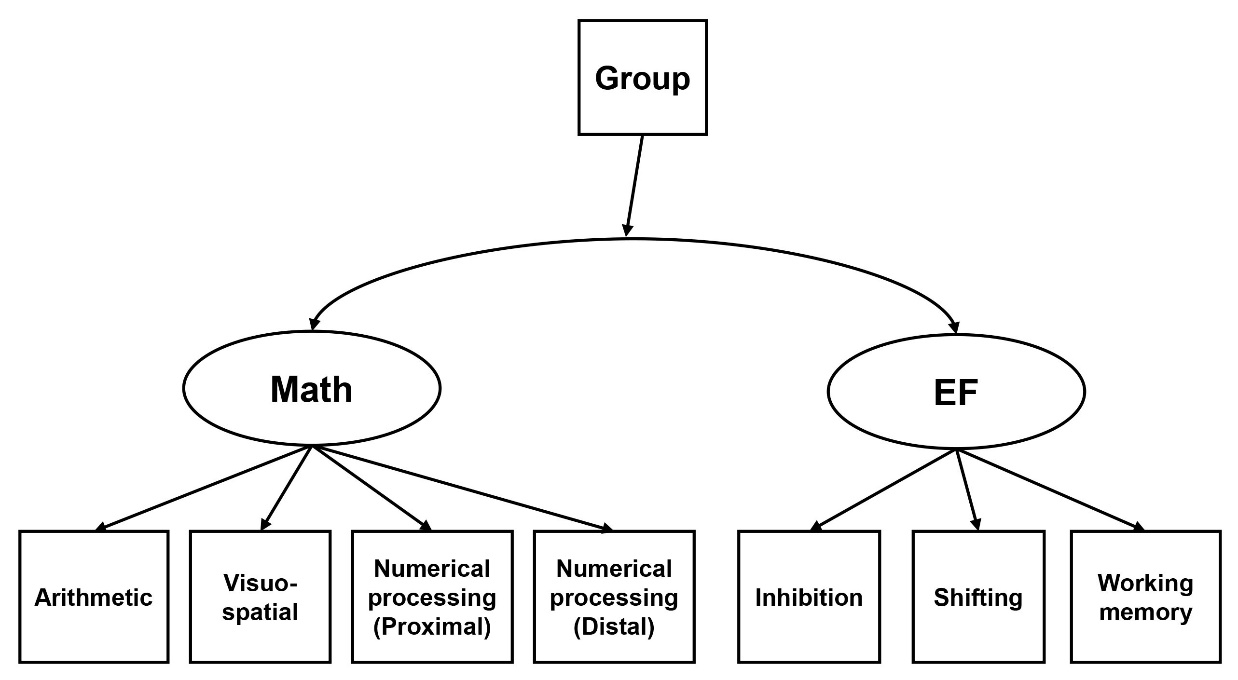


**Figure S6.** Schematic representation of the SEM evaluating AMC training vs. control group differences on the relationship between mathematical ability and EF skills. Note that group was not included as a dummy variable as the figure might suggest, but the correlated factor model was applied simultaneously to the correlation matrices of the two groups in the multi-group SEM framework. This visualization has been chosen to illustrate more simply that the multi-group model allows testing whether the correlation between the two latent variables differs between groups.

**Supplementary Tables**

**Table S1.** Demographic characteristics of post-training stage.


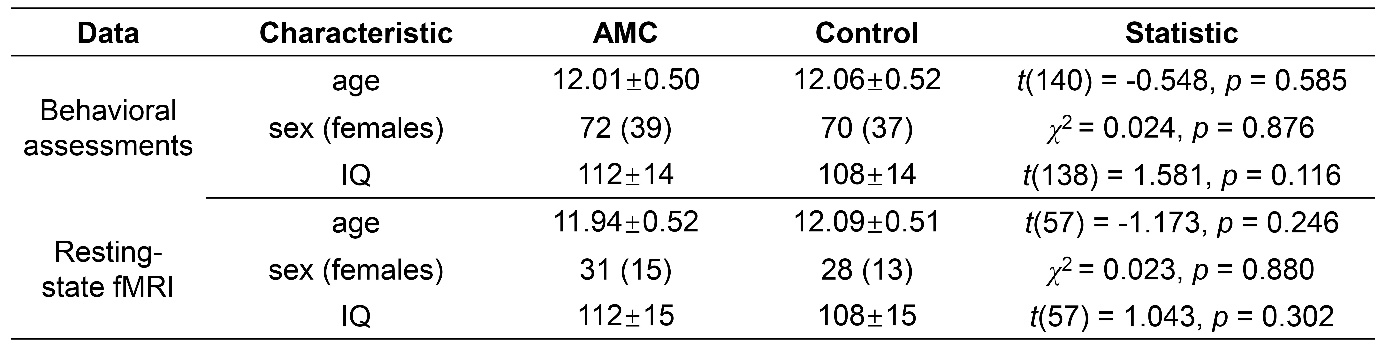


**Table S2.** Group matching on demographic and cognitive variables in baseline.


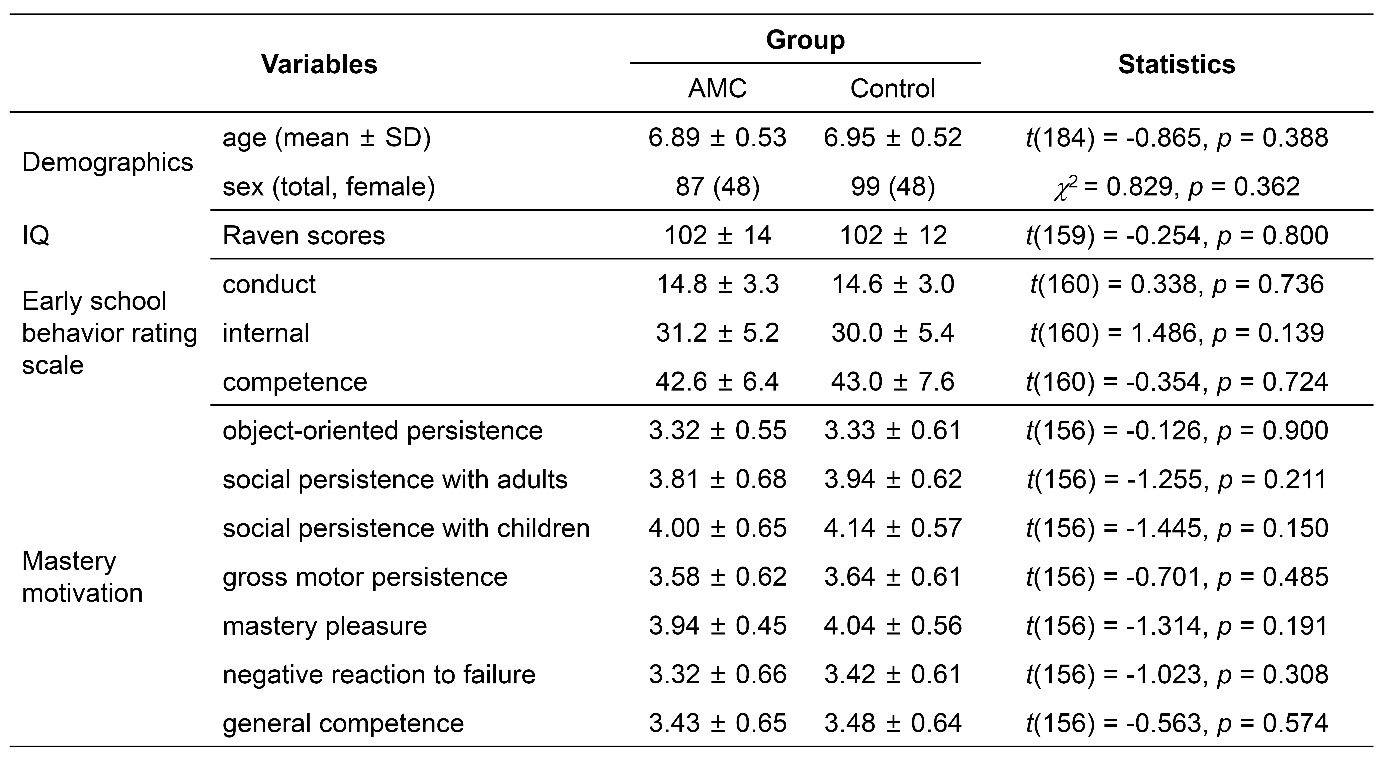


**Table S3.** Standard Mental Abacus Level Test.


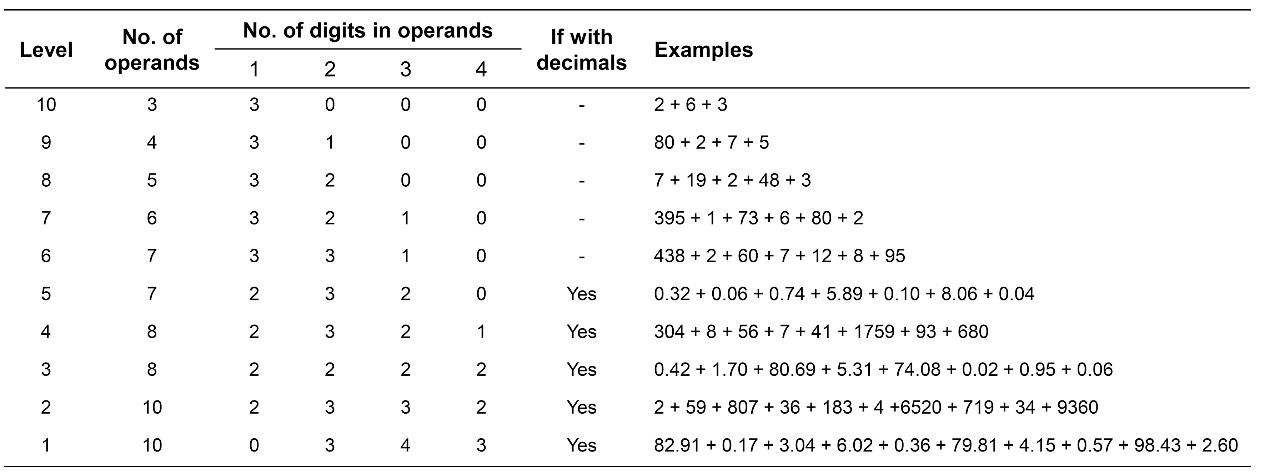


Note: This assessment evaluates the mental abacus proficiency of children in the training group, with Level 1 representing the highest achievable performance tier. This full test (over Level 7) also includes the multiplication and division parts, which require the participants to complete the whole test using abacus-based mental calculation within a limited time.
